# Lipoplex-based targeted gene therapy for the suppression of tumours with VEGFR expression by producing anti-angiogenic molecules

**DOI:** 10.1186/s12951-020-00610-9

**Published:** 2020-04-09

**Authors:** Shu-Yi Ho, Pin-Rong Chen, Chia-Hung Chen, Nu-Man Tsai, Yu-Hsin Lin, Chen-Si Lin, Cheng-Hsun Chuang, Xiao-Fan Huang, Yi-Lin Chan, Yen-Ku Liu, Chen-Han Chung, Shun-Long Weng, Kuang-Wen Liao

**Affiliations:** 1 Department of Biological Science and Technology, National Chiao Tung University, Hsinchu City, 30068 Taiwan, ROC; 2 Institute of Molecular Medicine and Bioengineering, National Chiao Tung University, Hsinchu City, 30068 Taiwan, ROC; 3 Department of Medical Research, Hsinchu Mackay Memorial Hospital, Hsinchu City, 30071 Taiwan, ROC; 4 Department of Medical Laboratory and Biotechnology, Chung Shan Medical University, Taichung City, 40201 Taiwan, ROC; 5 Department of Pathology and Clinical Laboratory, Chung Shan Medical University Hospital, Taichung City, 40201 Taiwan, ROC; 6 Ph.D. Program in Industrial Development of College of Biological Science and Technology, National Chiao Tung University, Hsinchu City, 30068 Taiwan, ROC; 7 Department of Veterinary Medicine, School of Veterinary Medicine, National Taiwan University, Taipei City, 10617 Taiwan, ROC; 8 Animal Cancer Center, College of Bioresources and Agriculture, National Taiwan University, Taipei City, 10617 Taiwan, ROC; 9 Institute of Medicine of Chung, Shan Medical University, Taichung City, 40201 Taiwan, ROC; 10 Department of Life Science, Chinese Culture University, Taipei City, 11114 Taiwan, ROC; 11 Hank Clinic Orthopedics Surgery, Miaoli County, 35157 Taiwan, ROC; 12 Department of Medicine, MacKay Medical College, New Taipei City, 25245 Taiwan, ROC; 13 Department of Obstetrics and Gynecology, Hsinchu MacKay Memorial Hospital, Hsinchu City, 30071 Taiwan, ROC; 14 Graduate Institute of Medicine, College of Medicine, Kaohsiung Medical University, Kaohsiung City, 80708 Taiwan, ROC; 15 Center for Intelligent Drug Systems and Smart Bio-Devices, National Chiao Tung University, Hsinchu City, 30068 Taiwan, ROC

**Keywords:** LPPC, Gene therapy, Anti-angiogenesis, RBDV, VEGFR

## Abstract

**Background:**

The anti-angiogenic fusion protein RBDV-IgG1 Fc (RBDV), which comprises the receptor-binding domain of vascular endothelial growth factor-A (VEGF-A), has shown antitumour effects by reducing angiogenesis in vivo. This study used the cationic lipoplex lipo-PEG-PEI-complex (LPPC) to simultaneously encapsulate both the RBDV targeting protein and the RBDV plasmid (pRBDV) without covalent bonds to assess VEGFR targeting gene therapy in mice with melanoma in vivo.

**Results:**

LPPC protected the therapeutic transgene from degradation by DNase, and the LPPC/RBDV complexes could specifically target VEGFR-positive B16-F10 cells both in vitro and in vivo. With or without RBDV protein-targeting direction, the pRBDV-expressing RBDV proteins were expressed and reached a maximal concentration on the 7th day in the sera after transfection in vivo and significantly elicited growth suppression against B16-F10 melanoma but not IgG1 control proteins. In particular, LPPC/pRBDV/RBDV treatment with the targeting molecules dramatically inhibited B16-F10 tumour growth in vivo to provide better therapeutic efficacy than the treatments with gene therapy with IgG1 protein targeting or administration of a protein drug with RBDV.

**Conclusions:**

The simultaneous combination of the LPPC complex with pRBDV gene therapy and RBDV protein targeting might be a potential tool to conveniently administer targeted gene therapy for cancer therapy.

## Background

As the sizes of tumours increase to more than 1–2 mm^3^, the microenvironments of the tumour will become hypoxic to threaten tumour growth. At this time, the tumours will disrupt the balance between pro- and anti-angiogenic factors within the microenvironment of tumour areas to facilitate angiogenesis [1, 2]. Under such conditions, various pro-angiogenic factors, including growth factors and proinflammatory cytokines, increase their expression to promote angiogenesis, which contributes to tumour growth, persistence, and metastasis [3–5]. Without such angiogenesis, the tumours will undergo necrosis [6].

Thus, interference in the VEGF-VEGFR axis signalling pathway to inhibit angiogenesis has been under development to suppress both tumour growth and metastasis due to all of the angiogenic factors, with VEGF playing the most crucial roles [7–10]. For tumour therapy, bevacizumab [an anti-VEGF humanized monoclonal antibody (mAb)], aflibercept (an anti-VEGF fusion protein) and ramucirumab (an anti-VEGFR-2 human mAb) have been developed and shown to inhibit the VEGF-VEGFR interaction and indeed provides an excellent therapeutic effect in patients with tumours [11–13] and in experimental animal models [14–16]. However, certain obstacles exist in the clinical trials of anti-angiogenic protein-based therapies. First, some acute and unusual toxicities have been observed, including gastrointestinal perforation and arterial thromboembolic complications [17–19]. Second, clinical results show that protein drugs need repeated administration to maintain a therapeutic concentration in tissues due to their relatively short half-lives. Third, pharmacokinetic studies have also shown that the administration of therapeutic proteins might not be optimal in the body, as they cannot maintain a continuous stable elevated level [20–22]. Therefore, high-dose administration of therapeutic proteins is required for a good therapeutic effect, especially for anti-angiogenesis proteins. Finally, the prices for the production and purification of protein drugs still cannot be lowered, and protein drugs are more expensive than traditional chemo drugs, which causes an economic burden.

Therefore, gene therapy for the continued expression of anti-angiogenic proteins has become an attractive approach, in which non-viral vectors may provide several advantages, such as being non-pathogenic, less immunogenic, not limited to transgene size, of low cost, and simple to prepare [23–25]. Within the non-viral gene delivery system, lipoplexes have become popular for cancer gene therapy. Moreover, lipoplexes are modified with various targeting tools to specifically deliver a drug to its target [26–31].

In cancer, the difference in the densities of endothelial cells between tumour tissues and normal tissues may be 50-fold, and the density could be a tumour-specific target that makes it easily accessible for drug administration [32]. RBDV-IgG1 Fc (RBDV), a recombinant fusion protein constructed by the receptor-binding domain of VEGF-A and the Fc fragment of human IgG1, can suppress tumour growth and angiogenesis in C57BL/6 mice after administration [33].

However, using RBDV as an anti-angiogenic protein also has its drawbacks, similar to other anti-angiogenic protein-based therapies. Hence, we used the cationic lipoplex (LPPC) as the vector for targeted gene therapy. LPPC is composed of two polymers, polyethylene glycol (PEG) and polyethylenimine (PEI), and two lipids, 1,2-Dioleoyl-*sn*-glycero-3-phosphocholine (DOPC) and 1,2-dilauroyl-*sn*-glycero-3-phosphocholine (DLPC). It has been shown that LPPC can be complexed with proteins in a non-covalent linkage manner, and the bound proteins which still retain their biological activity cannot be displaced by unbound proteins [34, 35]. With this property, LPPC has been used as a vector for targeted tumour therapy in combination with Herceptin [36]. However, the use of LPPC for targeting gene therapy has been still unknown.

In this study, LPPC simultaneously encapsulated an RBDV plasmid (pRBDV) and targeted the RBDV protein to achieve specific targeted gene therapy for melanoma. The results showed that LPPC-based gene therapy could contribute to a better therapeutic efficacy than administration of protein drugs alone.

## Results

### The characteristics of the LPPC/DNA complexes

**Fig. 1:**
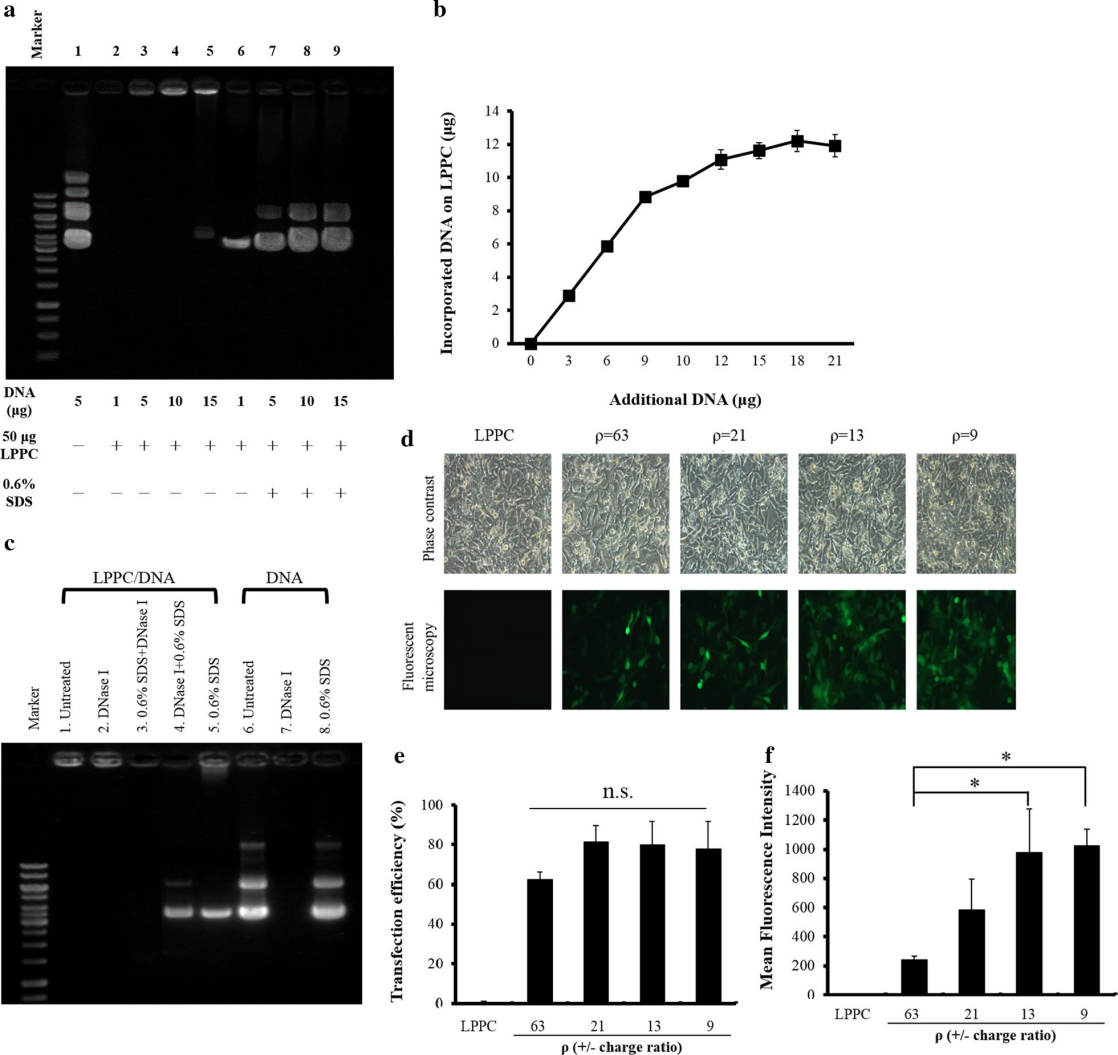
Characteristics of the LPPC/DNA complexes **a** LPPC (50 μg) was incubated with different amounts of DNA for 30 min at room temperature, and the complexes were determined by 0.8% agarose electrophoresis. Lane 1 was 5 μg DNA alone and lanes 2–5 were 1, 5, 10, and 15 μg DNA with LPPC, respectively. The replacement of DNA from the complexes by competition with 0.6% SDS is shown in lanes 6–9. **b **Different amounts of DNA were incubated with 50 μg of LPPC for 30 min at room temperature, and the amounts of bound DNA were analysed with a spectrophotometric assay. **c** The ability of LPPC to protect DNA from DNase I digestion was assessed by treatment with DNase I. Lane 1 was the LPPC/DNA complexes alone; lanes 2–5 were LPPC/DNA complexes treated with DNase I, SDS+ DNase I, DNase I+ SDS and SDS alone, respectively; and lanes 6–8 were DNA alone, DNA treated with DNase I, and DNA treated with SDS, respectively. **d** Different amounts of pAAV-MCS-hrGFP plasmids with 50 μg of LPPC at ρ = 63, 21, 13, 9 (± charge ratio) were transfected into B16-F10 cells, and the cells were observed under a microscope after 48 h. **e**, **f** Flow cytometry was used to analyse the transfection efficacy and the mean fluorescence intensity. The data represent the mean ± SD (n = 6). Significant differences were evaluated by ANOVA with the Bonferroni test and labelled as *P < 0.05

The gel retardation assay showed that the LPPC particles encapsulated different amounts of plasmid DNAs to form complexes and are trapped in the loading wells of the agarose gel (Fig. 1a, lanes 2–5). Furthermore, 0.6% SDS disrupts the liposomal complex and releases plasmid DNAs, which are visible as bands on the gel (Fig. 1a, lanes 6–9). Additionally, the maximal DNA binding capacity of 50 μg LPPC was approximately 12 μg (Fig. 1b). Moreover, the particle diameters or surface charges of different LPPC complexes were examined by dynamic light scattering. Table 1 shows that the average size of LPPC is 197.1 ± 17.8 nm, and the binding of DNA, protein, or PEG to LPPC increases the particle size of LPPC from 197.1 ± 17.8 to 527.5 ± 83.4 nm. In addition, the surface charge of the LPPCDNA complexes and LPPC/RBDV complexes decreased from 43.2 ± 1.4 to 4.9 ± 2.0 mV.

**Table 1:** Average diameters (nm) and zeta potentials (mV) of LPPC complexes (per 50 μg of LPPC)

Formulation	Size (nm)	Zeta potential (mV)
LPPC	197 ± 17.8	4302 ± 1.4
LPPC + 1 µg DNA	263.1 ± 27.9	37.4 ± 1.5
LPPC + 3 µg DNA	287.7 ± 29.9	34.9 ± 2.4
LPPC + 5 µg DNA	320.4 ± 12.4	27.2 ± 4.8
LPPC + 7 µg DNA	365 ± 22.6	21.7 ± 3.4
LPPC + 5 µg DNA + 1 mg RBDV protein	426.7 ± 22.7	17.1 ± 2.3
LPPC + 5 µg DNA + 1 mg RBDV protein + PEG	527.5 ± 83.4	4.9 ± 2.0

The data represent the mean ± SD (n = 6)

The stability of the LPPC/DNA complex was examined for DNase I degradation, and the results indicate that DNase I can fully degrade the DNA released from the LPPC complexes but not the DNAs on the LPPC complexes (Fig. 1c, lanes 3–4). These results reveal that LPPC protects DNAs from DNase I degradation. Moreover, the LPPC complexes that inserted hrGFP plasmids can transfect B16-F10 cells, and the transfectant can express green fluorescence in a dose-dependent manner (Fig. 1d). Furthermore, the fluorescence of these transfectants was determined by flow cytometry to demonstrate the efficiency and expression of the transfection. The results show that LPPC can transfect the plasmid DNAs into the cells, and the transfectants can express the transgene in a dose-dependent manner (Fig. 1e, f).

### In vitro targeting activity of RBDV-IgG1 Fc (RBDV) on LPPC to VEGFR-positive cells

**Fig. 2:**
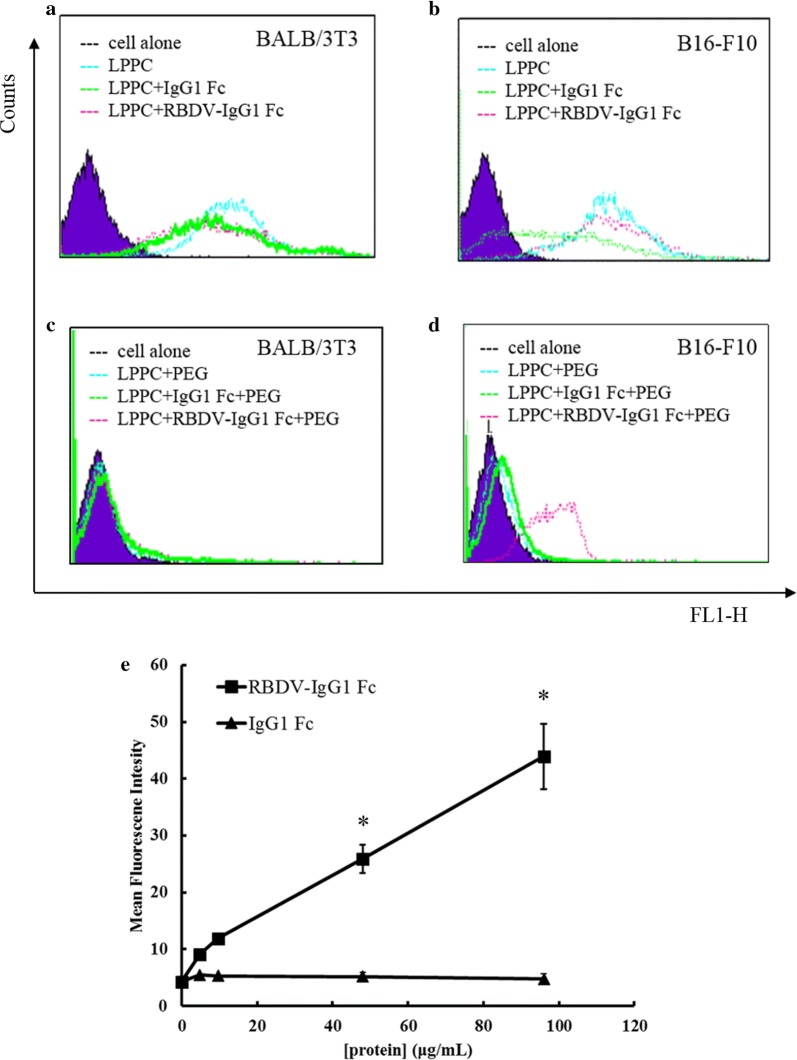
The binding activities of the LPPC/DiO/RBDV-IgG1 Fc complexes. **a** BALB/3T3 cells and **b** B16-F10 cells were stained with DiO-labelled LPPC complexes without PEG 1.500 complexed. **c** BALB/3T3 cells and **d** B16-F10 cells were stained with PEG-complexed LPPC/DiO complexes. **e** DiO-labelled LPPC complexes (50 μg) were incubated with different amounts of RBDV-IgG1 Fc or IgG1 Fc proteins for 30 min, and then the complexes were added to B16-F10 cells. The binding intensity was analysed by flow cytometry. The data represent the mean ± SD (n = 6). Significant differences are evaluated by Student’s *t*-test and are labelled as *P < 0.05

RBDV was used to adsorb on DiO-labelled LPPC to evaluate the specific targeting activity to VEGFR-positive cells (B16-F10 cells) and VEGFR-negative cells (BALB/3T3 cells). Figure 2a, b show that the DiO-labelled LPPC with or without RBDV or IgG1 Fc (negative control protein) all have a high fluorescence intensity in both cell lines compared to the untreated cells, which suggests that the cationic character of LPPC causes nonspecific binding of the cells. To improve the specific targeting of the liposomal particles, PEG was used to attenuate the extra cationic charges of the LPPC/protein complex. With PEG complexing, the LPPC/RBDV/PEG complex lost its non-specific binding activity to BALB/3T3 cells but still maintained binding activity to B16-F10 cells; meanwhile, LPPC/PEG and LPPC/IgG1 Fc/PEG also lowered their non-specific binding activities in both cell lines (Fig. 2c, d). Figure 2e shows that the targeting ability of DiO-labelled LPPC to B16-F10 cells by RBDV is dose-dependent.

**Fig. 3:**
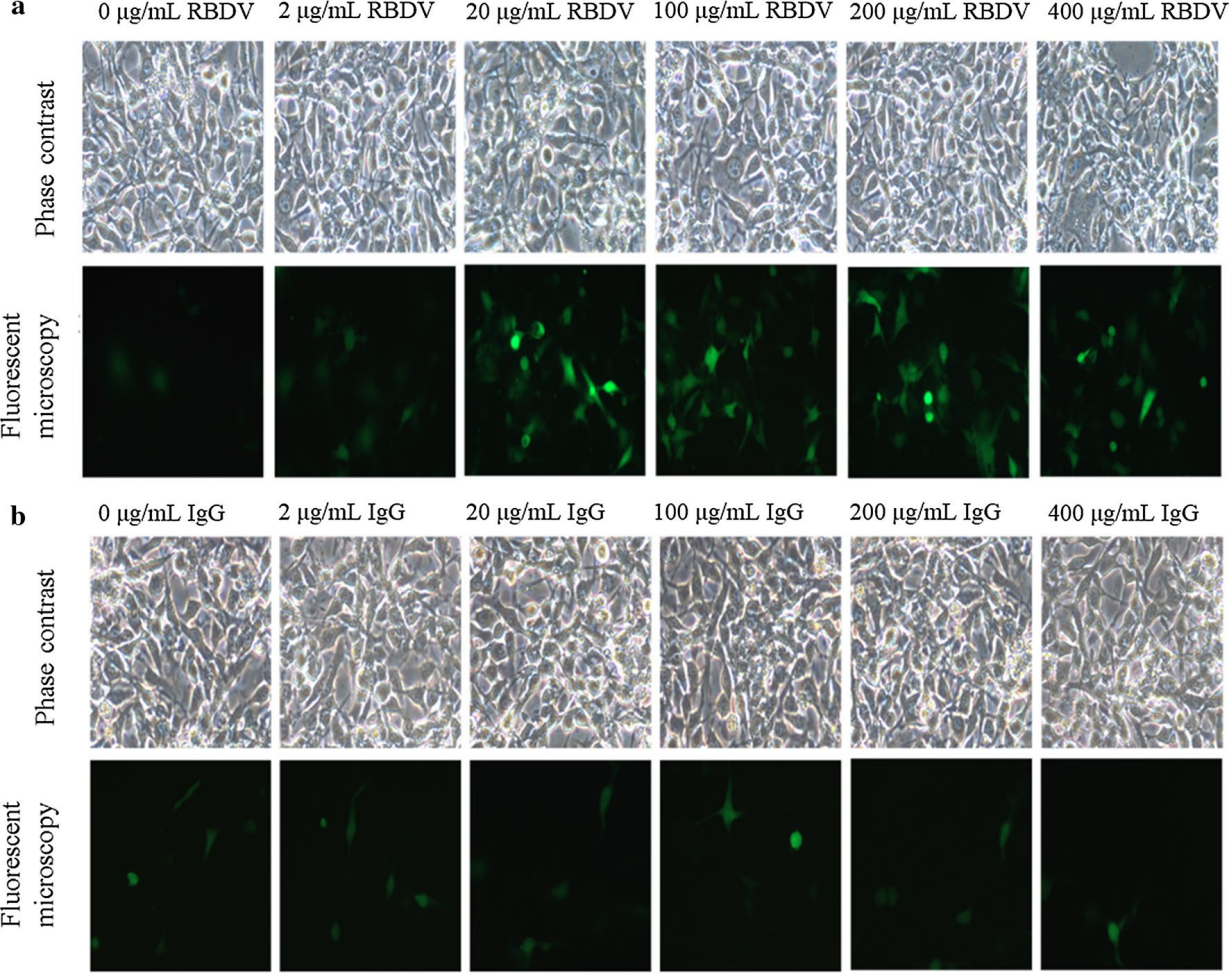
In vitro transfection efficiency of the LPPC/DNA/RBDV complexes. Different concentrations of **a** RBDV complexes or **b** IgG1 Fc with 5 μg of DNA and 50 μg of LPPC were transfected into B16-F10 cells, and the cells were observed under a microscope 48 h after transfection. LPPC, which encapsulated with RBDV or IgG1 Fc, were all complexed by PEG

The abilities of LPPCs to encapsulate different amounts of RBDV or IgG1 Fc proteins for transfection were examined, and the results revealed that the transfectant expressed high fluorescence after treatment with LPPC with different amounts of RBDV in a dose-dependent manner but only showed a low fluorescent signal after treatment with IgG1 Fc (Fig. 3a, b). Flow cytometry also demonstrated that RBDV enhanced the transfection efficiency and the expression of the transfection in a dose-dependent manner (Additional file 1: Figure S1).

### The effects of the RBDV protein expressed by the transfectant in B16-F10 cells

**Fig. 4:**
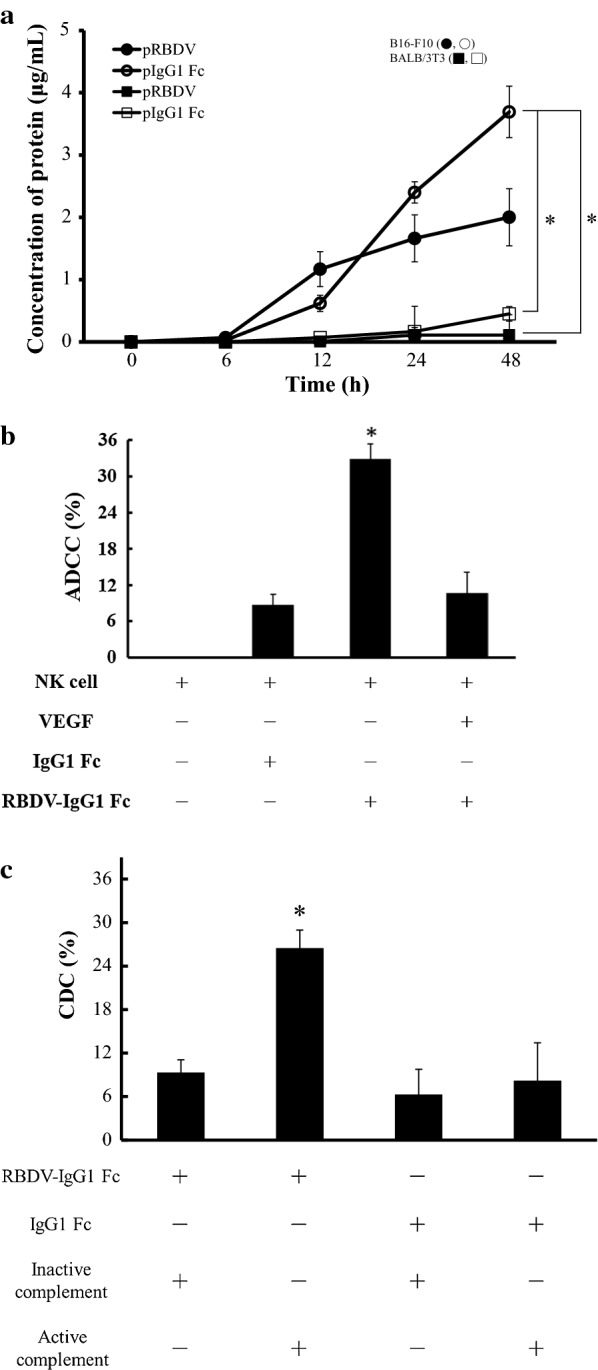
The expression of the RBDV protein and the in vitro cytotoxicity assay. **a** pRBDV or pIgG1 Fc were transfected into B16-F10 cells and BALB/3T3 cells. The black or white circles mean transfections of LPPC/pRBDV/RBDV/PEG or LPPC/pIgG1 Fc/RBDV/PEG in B16-F10 cells, respectively. The black or white squares mean transfections of LPPC/pRBDV/RBDV/PEG or LPPC/pIgG1 Fc/RBDV/PEG in BALB/3T3 cells, respectively. The cell culture media were analysed at 0, 6, 12, 24, and 48 h for the amount of protein expression by ELISA. LPPC, which encapsulated with RBDV, were all complexed by PEG. The data represent the mean ± SD (n = 3). Significant differences were evaluated by ANOVA with the Games-Howell test and labelled as *P < 0.05. **b** NK-92MI cells (effector cells) mixed with B16-F10 cells (target cells) at an E/T ratio of 1:1 were co-incubated with IgG1 Fc, RBDV-IgG1 Fc, or VEGF plus RBDV-IgG1 Fc for 5 h, and the cytotoxic activity was determined by MTT assay. The data represent the mean ± SD (n = 6). Significant differences were evaluated by ANOVA with the Bonferroni test and labelled as *P < 0.05 compared with other groups. **c** Complement mixed with B16-F10 cells was co-incubated with IgG1 Fc or RBDV-IgG1 Fc, and the cytotoxic activity was determined by MTT assay. The data represent the mean ± SD (n = 4). Significant differences were evaluated by ANOVA with the Bonferroni test and labelled as *P < 0.05 compared with other groups

Figure 4a shows the expressive abilities of the transfectants after LPPC with RBDV and different plasmids at different times. The VEGFR-positive transfectants significantly expressed RBDV or IgG1 Fc after transfection with pRBDV or pIgG1 Fc. As previously described, VEGFR-negative cells (BALB/3T3 cells) express few or no recombinant proteins at any time after transfection (Fig. 4a). Then, the recombinant proteins in the culture supernatants were examined for their bioactivities. Figure 4b, c show that the pRBDV-expressed RBDV proteins in the B16-F10 transfectants can significantly elicit ADCC and CDC effects against B16-F10 cells, while the control protein, IgG1 Fc, cannot.

### Targeted transfection ability of the LPPC/RBDV complexes in vivo

**Fig. 5:**
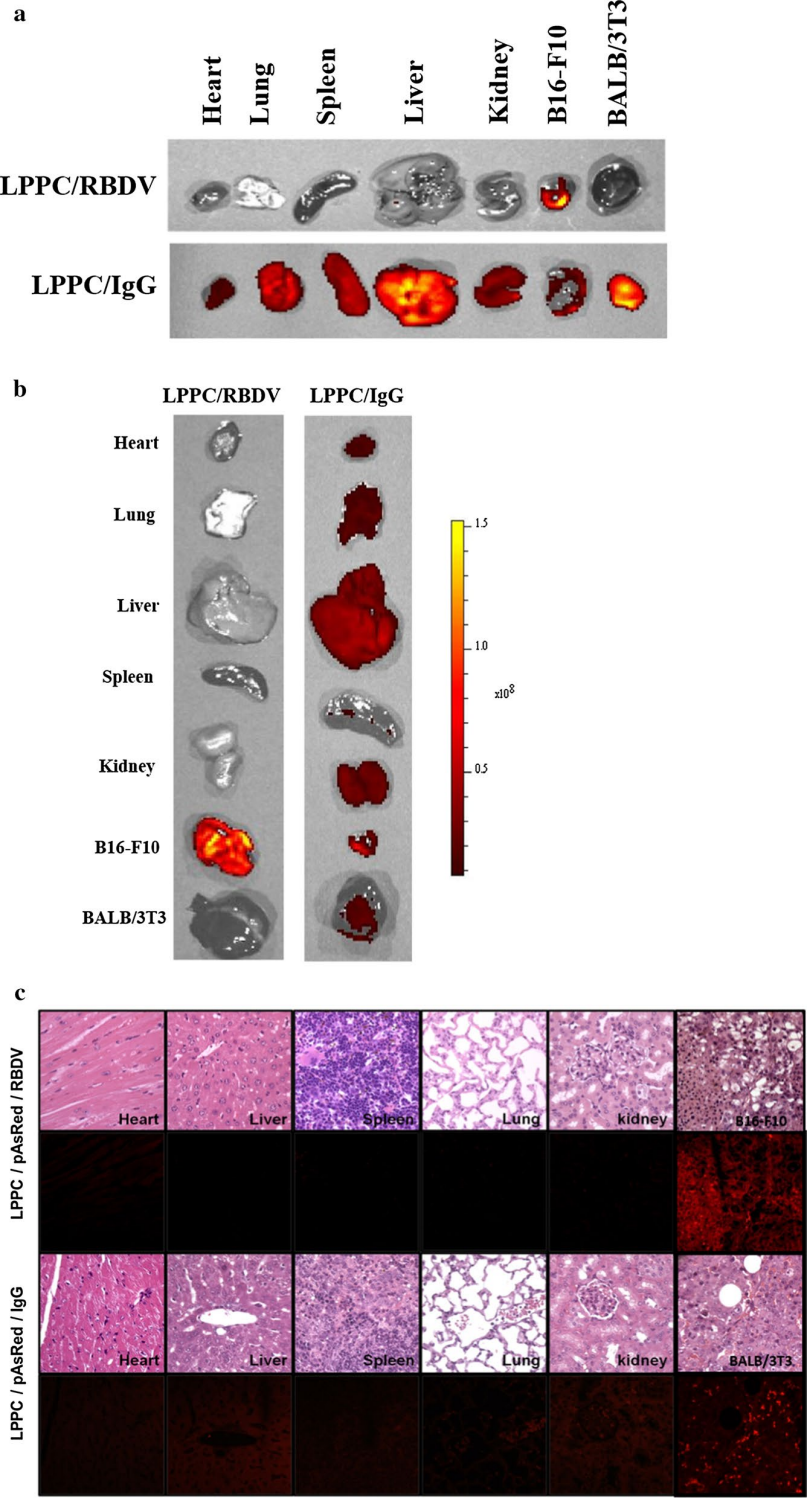
Targeting ability and Targeted transfection ability of the LPPC/RBDV complexes in vivo. **a **Tumour-bearing mice were i.v. injected with LPPC/DiO/RBDV or LPPC/DiO/IgG1 Fc. After 72 h, the organs and tumours were excised and imaged with an IVIS system at the appropriate wavelength (Em: 600 nm and Ex: 465 nm). **b** Tumour-bearing mice were i.v. injected with LPPC/pAsRed/RBDV or LPPC/pAsRed/IgG1 Fc. After 6 days, the organs and tumours were excised and imaged with an IVIS system at the appropriate wavelength (Em: 560 nm and Ex: 465 nm). **c** The tumours and organs were further processed by H&E staining and photographed with a fluorescence microscope. LPPC, which encapsulated with RBDV or IgG1 Fc, were all complexed by PEG

The IVIS imaging system first examined the in vivo targeting ability of DiO-labelled LPPC to determine the effects of RBDV and IgG1 Fc. The results show that the RBDV/DiO-labelled LPPC complexes can target B16-F10 tumours at 72 h and do not accumulate in BALB/3T3 cells or other organs (Fig. 5a). Then, the transfection ability of the LPPC/RBDV complexes was assessed by carrying pAsRed2-N1 to observe the expression of red fluorescent proteins in vivo. Figure 5b shows that the reporter gene can be significantly expressed in B16-F10 tumours but not in other organs after 6 days. In addition, the tissue sections also revealed that with RBDV, LPPC/pAsRed2-N1 encoded red fluorescent proteins in B16-F10 tumour tissues but not in other organ tissues. Conversely, the IgG-absorbed LPPC/pAsRed2-N1 lost its ability to be specifically expressed in tumour tissues and caused nonspecific expression in heart, liver, spleen, lung, and kidney tissues (Fig. 5c).

### Inhibition of B16-F10 tumour growth by the expressed RBDV protein

**Fig. 6:**
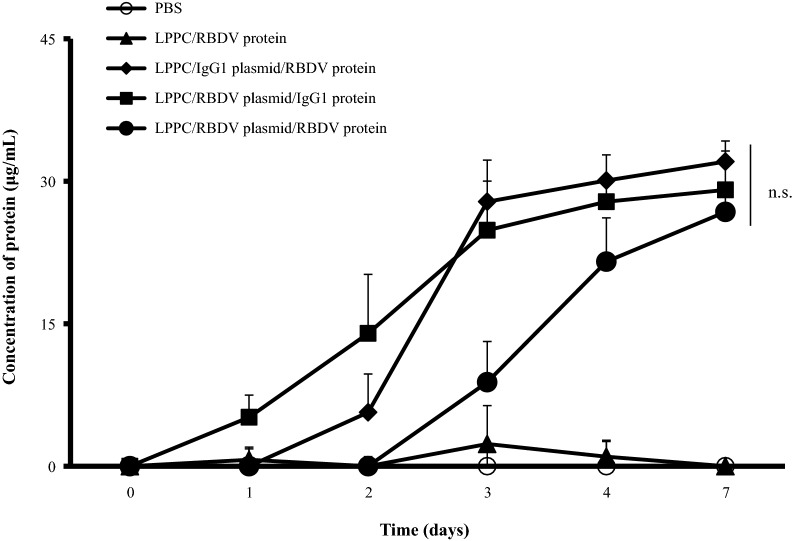
In vivo expression of RBDV or IgG1 Fc. The LPPC/RBDV-IgG1 Fc protein complexes, LPPC/IgG1 Fc plasmid/RBDV-IgG1 Fc protein complexes, LPPC/RBDV-IgG1 Fc plasmid/IgG1 Fc protein complexes, LPPC/RBDV-IgG1 Fc plasmid/RBDV-IgG1 Fc protein complexes and PBS were i.v. injected into mice. Mouse sera were collected at different times post-administration and analysed by ELISA. LPPC, which encapsulated with RBDV or IgG1 Fc, were all complexed by PEG. The data represent the mean ± SD (n = 3). Significant differences were evaluated by ANOVA with the Bonferroni test

**Fig. 7:**
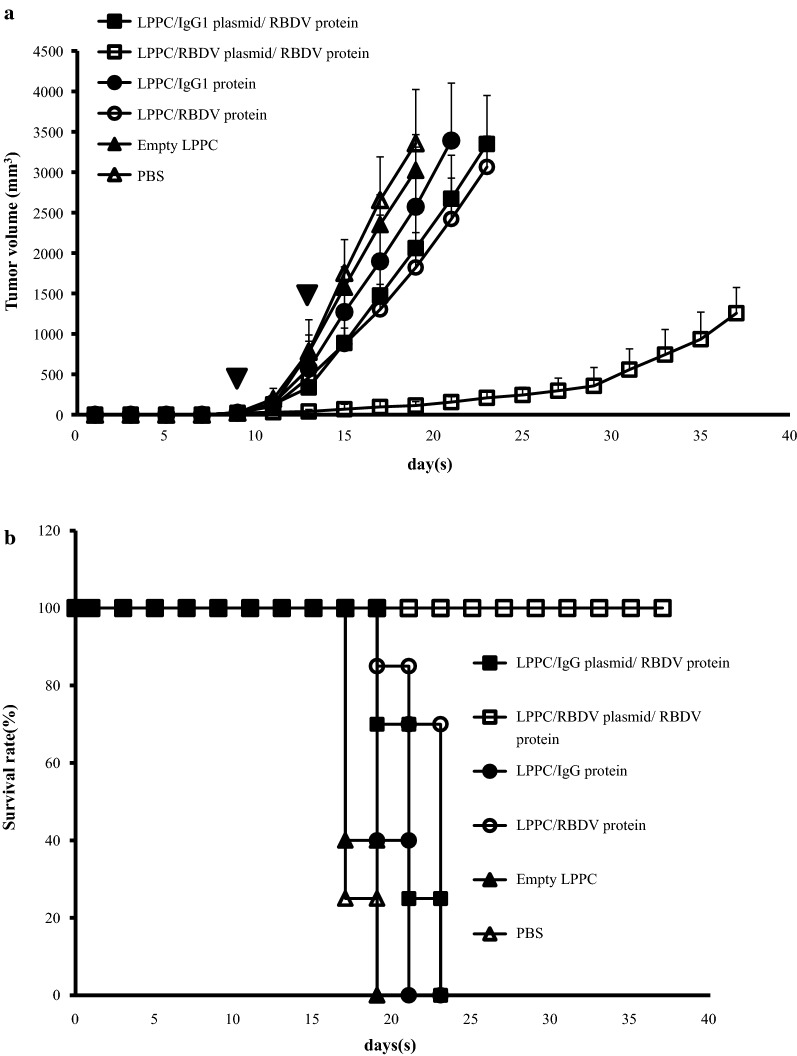
Inhibition of tumour growth by the LPPC/RBDV-IgG1 Fc plasmid/RBDV-IgG1 Fc protein. **a** Female C57BL/6 mice (6–8 weeks of age) were subcutaneously inoculated with 1 × 10^6^ cells in 100 mL of PBS. When the average tumour volume reached 30 mm^3^, the mice were i.v. injected with LPPC/RBDV-IgG1 Fc plasmid/RBDV-IgG1 Fc protein or other groups and injected again after four days. Inverted filled triangle means the day of complex injection. Tumour volume was measured every 2 days after injecting the complexes, and the mice were sacrificed when the tumour grew to a size of 2500 mm^3^. The data represent the mean tumour volume ± SD (n = 7). **b** The survival rate of C57BL/6 mice bearing B16-F10 tumours treated with LPPC/RBDV-IgG1 Fc plasmid/RBDV-IgG1 Fc protein or other groups. LPPC, which encapsulated with RBDV or IgG1 Fc, were all complexed by PEG

**Fig. 8:**
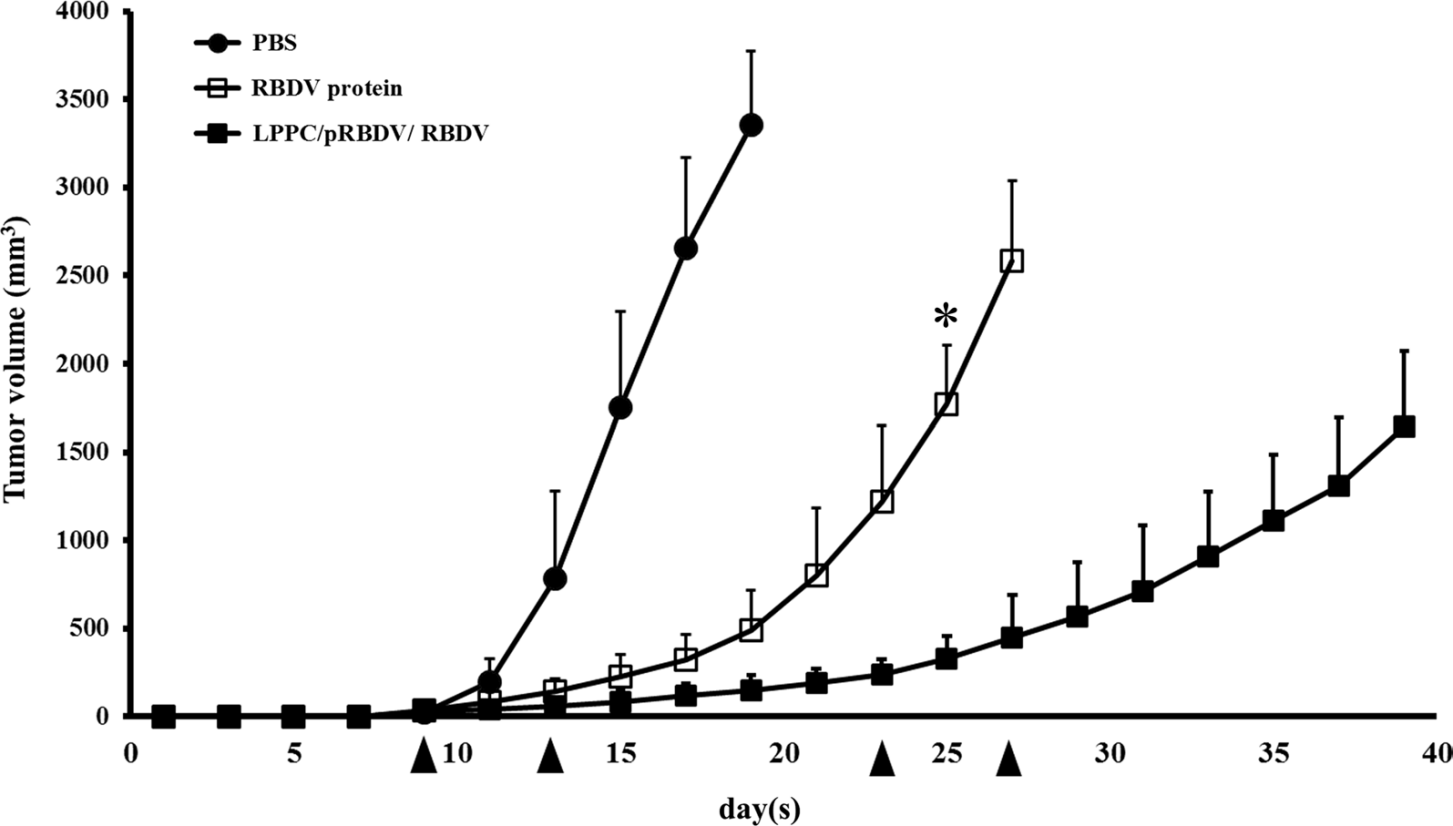
Inhibition of tumour growth by LPPC/pRBDV/RBDV and the RBDV protein. Female C57BL/6 mice (6–8 weeks of age) were subcutaneously inoculated with 1 × 10^6^ cells in 100 mL of PBS. When the average tumour volume reached 30 mm^3^, the mice were i.v. injected with LPPC/pRBDV/RBDV or RBDV protein. Inverted filled triangle means the day of complex injection. Tumour volume was measured every 2 days after injection of the complexes, and the mice were sacrificed when the tumour grew to a size of 2500 mm^3^. LPPC, which encapsulated with RBDV or IgG1 Fc, were all complexed by PEG. The data represent the mean ± SD (n = 7). Significant differences are evaluated by Student’s *t*-test and are labelled as *P < 0.05

The in vivo expression of LPPC-mediated transgenes was followed by intravenous (i.v.) injection with different formulas, and the sera of the treated mice were collected and measured. Figure 6 indicates that the LPPC/plasmids with either RBDV or IgG1 Fc can transfect the transgene and express the recombinant proteins after i.v. injection for 7 days. Thus, the effects of one i.v. injections with different LPPC complexes on B16-F10 tumour growth were examined. The results show that LPPC-mediated transfections significantly inhibited tumour growth compared with PBS-treated groups over 17 days. The order of the inhibitory efficacy for the treatments was LPPC/pRBDV/RBDV > LPPC/pRBDV/IgG1 > LPPC/RBDV = LPPC/pIgG1/RBDV (Additional file 1: Figure S2). To further assess the therapeutic effects of LPPCs on B16-F10 tumour growth, mice were i.v. injected twice with distinct LPPC complexes. The results show that only the LPPC/pRBDV/RBDV treatment dramatically inhibited tumour growth compared with the other groups (Fig. 7a); moreover, treatment with LPPC/pRBDV/RBDV significantly improved the survival time compared with the other groups (Fig. 7b). Furthermore, the therapeutic effects of LPPC/pRBDV/RBDV were compared with the effects of recombinant RBDV proteins after multiple injections. After four treatments, LPPC/pRBDV/RBDV had better tumour growth inhibition than the recombinant RBDV protein alone (Fig. 8).

## Discussion

In this study, LPPC showed excellent gene transfection capability both in vitro (Fig. 1d) and in vivo (Fig. 5b, c). In combination with targeting molecules, LPPC has been shown to be a specific delivery vector for an antitumour drugs to tumour cells [36]. The targeting molecule RBDV-IgG1 Fc (RBDV) further causes specific gene transfection in vitro (Fig. 3) and in vivo (Fig. 5b, c), which all make LPPC a novel vector in gene therapy. On the other hand, pRBDV also shows benefits of gene therapy on antitumour effects by expressing a therapeutic molecule in vivo (Figs. 5b, c and Additional file 1: Figure S2) through the mechanisms of ADCC and CDC in vitro (Fig. 4b, c). Moreover, we have also confirmed that RBDV protein-directed gene therapy of pRBDV can dramatically suppress tumour progression in mouse melanoma in vivo and provides a better therapeutic efficacy than the administration of pure RBDV protein (Fig. 8) in addition to being a target molecule.

It is an original strategy to use a therapeutic gene that expresses the same therapeutic protein as the targeting molecule (RBDV) at the same time. Currently, researchers have designed a strategy for specific targeted gene therapy using a therapeutic transgene expressing a therapeutic protein that is different from the targeting molecule. For example, scholars have used a PEI-PEG copolymer to covalently link to the anti-HER2 antibody for the enhancement of truncated-Bid (tBid) killer gene expression, which causes the death of HER2-positive breast cancer cells [37]. Likewise, Huang et al. constructed an immunoliposome-loaded endoglin single-chain antibody for enhancing the expression of the porcine α1,3GT gene to suppress lung cancer [29]. Additionally, Nusrat Khan and his co-workers developed a CD33 targeting vector to express an inducible caspase-9 suicide gene in acute myeloid leukaemia therapy [38]. None of the current studies of nanoparticle technology in cancer therapy have had the same idea as this study does, and this strategy indeed provides a better therapy than protein-targeted therapy.

In addition, using LPPC as a transfection reagent in this strategy also provides several advantages for specific gene therapy. First, the positive charge of PEI can bind DNA electrostatically and protect the binding DNA from DNase degradation (Fig. 1c). Second, LPPC can stably adsorb proteins via a noncovalent linkage, which retains the flexibility to encapsulate different targeted proteins without damaging the activity of the targeting molecules. Although the covalent linkage of the target molecules to liposomes is a prevalent method for conjugation, this process may attenuate the activities of specific targeting molecules [39, 40]. Third, LPPC can be centrifuged to form a pellet, which allows LPPC to be easily separated from the unincorporated molecules to avoid the interference of free proteins prepare for use.

Interestingly, although previous studies have shown that RBDV can provide a good anti-angiogenesis effect against tumour growth by targeting both VEGFR1 and VEGFR2, the ability to cause tumour regression by RBDV protein treatment is less useful than pRBDV in this study. Moreover, LPPC with both RBDV and pRBDV revealed the best therapeutic effects among all groups (Fig. 8). There are two possibilities that could explain such a phenomenon. First, RBDV can specifically bind to VEGFR1 and VEGFR2, which leads the LPPC complex to the tumour site and transfects pRBDV. Then, the newly encoded RBDV proteins will have a relatively higher concentration in the tumour microenvironment than that in other tissues, which contributes to the antitumour effects. Second, the pRBDV transfectant can continue expressing RBDV over time, which makes it a better therapeutic than RBDV.

## Conclusions

In conclusion, we show that LPPC could adsorb not only plasmid DNA and the RBDV protein but also retain their bioactivities. The LPPC/RBDV complexes could target B16-F10 tumours, and the reporter gene pAsRed2-N1 could be significantly expressed in B16-F10 tumours but not on other organs. Modification of cationic liposomes through the addition of specific targeting molecules may make it possible to increase uptake by angiogenetic endothelial cells and decrease uptake by healthy endothelial cells and macrophages. Hence, the concept of using LPPC-encapsulated pRBDV and the RBDV protein might be a useful strategy for specific gene delivery.

## Methods

### Cell lines, animals and reagents

Human embryonic kidney (HEK) 293T cells, mouse melanoma B16-F10 cells, and mouse embryonic fibroblast BALB/3T3 cells were obtained from the Bioresource Collection and Research Center (BCRC, Hsinchu City, Taiwan, ROC). These cells were cultured in Dulbecco’s modified Eagle’s medium (DMEM; Invitrogen, Gaithersburg, MD, USA) supplemented with inactivated 10% foetal bovine serum (FBS; Invitrogen) and 1% penicillin–streptomycin amphotericin B (PSA; Biological industries, New York, NY, USA). Human natural killer NK-92 MI cells were grown in alpha minimum essential medium (αMEM; Sigma-Aldrich, St. Louis, MO, USA) supplemented with 0.2 mM inositol (Sigma-Aldrich), 0.2 mM 2-mercaptoethanol (Sigma-Aldrich), 0.02 mM folic acid (Sigma-Aldrich), 12.5% horse serum (Gibco BRL, Gaithersburg, MD, USA) and 12.5% FBS. All cells were incubated at 37 °C in an atmosphere of 5% CO_2_.

Male C57BL/6 mice (6–8 weeks old) were purchased from the National Laboratory Animal Center (NLAC, Taipei City, Taiwan, ROC).

1,2-Dioleoyl-*sn*-glycero-3-phosphocholine (DOPC) (Avanti Polar Lipids, Inc., Alabaster, AL, USA), 1,2-dilauroyl-*sn*-glycero-3-phosphocholine (DLPC) (Avanti Polar Lipids, Inc.) and polyethylene glycol (PEG, MW 1.500) (Shimo-Meguro, Meguro-Ku, Tokyo, Japan), PEG (MW 8.000, Sigma-Aldrich) and polyethylenimine (PEI, branched, MW 25.000, Sigma-Aldrich) were purchased to prepare LPPC.

### Plasmid construction

The pAAV-MCS-hrGFP plasmid was cloned from pNF-kB-hrGFP and pAAV-MCS (Stratagene, La Jolla, CA, USA). The pAsRed2-N1 plasmid was purchased from BD Biosciences (San Jose, CA, USA). Plasmids pAAV-MCS/RBDV-IgG1 and pAAV-MCS/IgG1 were constructed according to the procedures in previous studies [33, 41]. All plasmids were amplified and purified with the Gene-Spin MidiPrep Kit (Protech Technology, Taipei City, Taiwan, ROC).

### Preparation of the RBDV-IgG1 Fc (RBDV) and IgG1 Fc recombinant proteins

RBDV and IgG1 Fc recombinant fusion proteins were produced and purified according to the procedures in previous studies [33, 41]. Briefly, the two plasmids were transfected into 293T cells. After 48 h, the supernatants of the cell culture media were collected and purified by Protein G-Agarose (Upstate, Inc., Lake Placid, NY, USA). Then, the eluted fractions were further purified by a nickel-charged HisTrap Hp affinity column (Amersham Biosciences, Piscataway, NJ, USA). Finally, the solvents of the eluted proteins were exchanged in PBS by a Sephadex G-25 prepacked column (Amersham Biosciences), and the recombinant fusion proteins were concentrated with Microcon Centrifugal Filters (Millipore, Bedford, MA, USA). The concentrations of the RBDV and IgG1 Fc recombinant fusion proteins were calculated by the Bradford assay by measuring the absorbance at 595 nm with a Sunrise^™^ absorbance microplate reader (TECAN, Männedorf, Zurich, Switzerland).

### Preparation of the LPPC and LPPC/DNA complexes

LPPC was produced according to the procedures in a previous study [34]. In brief, LPPC was prepared with two kinds of lipids (DOPC and DLPC) and two kinds of polymers (PEG and PEI). The molar ratio of lipids:PEI:PEG was approximately 13:5:5.

The amounts of DNA complexed to LPPC were based on the nitrogen/phosphate ratio (ρ), which refers to the molar ratio of amine and phosphate groups in PEI of LPPC and DNA, respectively. For LPPC/DNA complex preparation, 5 μL of LPPC (50 μg/mL) was mixed with 1, 3, 5, 7 μg of hrGFP plasmids at 25 °C for 30 min to give a final volume of 15 μL LPPC/DNA complexes at ρ = 63, 21, 13, 9, respectively.

For LPPC/DNA/RBDV complex preparation, 5 μL of LPPC (50 μg/mL) was mixed with 5 μg hrGFP plasmid at 25 °C for 30 min, and then different amounts of RBDV were added for another 30 min to a final volume of 50 μL LPPC/DNA/RBDV complexes. Finally, the extra positive charges of the complexes were attenuated with 50 μL of PEG 1.500 (100 mg/mL) for 30 min twice.

### Gel retardation assay

The binding of LPPC to the hrGFP plasmid with or without 0.6% SDS was assayed by gel retardation analysis. Different amounts of plasmids and 0.6% SDS were added to 50 μg of LPPC and briefly shaken. After 30 min of incubation at room temperature, 10 μL of the LPPC/DNA complexes were analysed by agarose gel electrophoresis. DNA was visualized with a UV lamp by using a Uni-photo gel image system (EZ lab, Keelung City, Taiwan, ROC).

### Size and zeta potential measurements

Ten microliters of the LPPC/DNA complexes were prepared as described above. The particle size and zeta potential were determined with a BI-200SM dynamic laser light scattering goniometer (Brookhaven, Inc., Holtsville, NY, USA).

### In vitro transfection

To study the transfection activity of the different LPPC/DNA complexes and LPPC/DNA/RBDV complexes, B16-F10 cells were seeded in 6-well plates at 2 × 10^5^ cells per well in 3 mL of DMEM containing 10% FBS and 1% PSA. The cell lines were incubated at 37 °C overnight in 5% CO_2_, and then the media was replaced with 1 mL of serum-free DMEM containing different LPPC complexes for 6 h at 37 °C. After 6 h, 2 mL of fresh DMEM growth media was added to the cells and followed by incubation for an additional 42 h. Then, the cells were resuspended in trypsin, and the transfection efficiency was measured by using FACScan flow cytometry (BD Biosciences).

### Antibody-dependent cell-mediated cytotoxicity (ADCC) and complement-dependent cytotoxicity (CDC) assay

The ADCC is typically triggered by immune cells such as NK cells, which can recognize the Fc region of IgG and induce the IgG-targeted cell to apoptosis. Besides, the CDC assay, which is triggered by the binding of C1q protein to the IgG can cause the IgG-targeted cell lysis by the activation of complement. Hence, the ADCC and CDC assays were used to verify the effects of RBDV at B16-F10 cells in presence of immunity in vitro.

Briefly, after 48 h of LPPC transfection, the supernatants of the cell culture media with the RBDV or IgG1 protein were collected for the analysis of ADCC and CDC assays.

In the ADCC assay, B16-F10 cells (target cells) were seeded in 96-well plates at 1 × 10^4^ cells per well overnight. Then, 2 μg of RBDV or IgG1 protein was added to the cells for 2 h, 1 μg/mL VEGF was added for 20 min, and the cells were treated with 2 μg of RBDV for another 1 h. NK-92 MI cells (effector cells) were also added to the plates at 1 × 10^4^ cells/well in 100 μL of αMEM for 4 h. Finally, the culture media was replaced by the working concentration of MTT (Sigma-Aldrich) for 4 h. The supernatants of the cell culture media were removed, and 100 μL of dimethyl sulfoxide (DMSO) was added to solubilize the MTT crystals. The ADCC effects on cell proliferation were determined by measuring the absorbance at 595 nm with a Sunrise^™^ absorbance microplate reader (TECAN).

In the CDC assay, B16-F10 cells were coated in a 96-well flat-bottom plate at 1 × 10^4^ cells per well. Two micrograms of RBDV or IgG1 protein was added to each well, and the cells were incubated for 2 h. Then, 200 μL of horse serum (Gibco BRL) as a source of active complement was added to each well for 2 h. Then, the supernatants of each well were removed and supplemented with MTT reagent for the cell proliferation assay.

### Quantification of the expressed RBDV and IgG1 Fc recombinant proteins

Plates were coated with 100 μL/well anti-human IgG antibody (250 ng) overnight at room temperature, followed by a blocking procedure with 2% skim milk in PBS for 1 h. RBDV and IgG1 Fc proteins ranging from 0 to 50 μg/mL were used to establish standard curves, and the supernatants from the in vitro transfection cell media or mouse sera were added. The standard ELISA protocol followed, and the absorbance was measured at 450 nm with a Sunrise absorbance microplate reader (TECAN).

### In vivo imaging

The tumour-targeting effects of RBDV were investigated with a Caliper IVIS Spectrum system (Caliper Life Sciences, Hopkinton, MA, USA), and the in vivo distribution of LPPC-modified complexes in C57BL/6 mice was observed. Briefly, B16-F10 or BALB/3T3 cells (1 × 10^5^) were implanted subcutaneously into the backs of mice. When the tumour size reached 50 mm^3^, two groups of mice were given either LPPC/DiO/RBDV or LPPC/DiO/IgG1 Fc in the amounts of 80 μg of RBDV, 80 μg of IgG1 Fc and 4 mg of LPPC via tail vein injection. At 0, 48, and 72 h post-injection, the mice were observed in the IVIS system with an appropriate wavelength of Em: 600 nm and Ex: 465 nm. Then, the mice were sacrificed, and the organs were imaged.

In another experiment, LPPC/pAsRed2N1/RBDV complexes were injected via the tail vein. At 6 days post-injection, the mice were sacrificed, and then the organs and tumours were excised and imaged with wavelengths Em: 560 nm and Ex: 465 nm.

### Therapeutic experiment

B16-F10 cells (1 × 10^5^) were implanted subcutaneously into the backs of mice. When the average tumour volumes reached 30 mm^3^, which occurred within 7 days post-implantation, the mice were injected intravenously with RBDV/pRBDV/LPPC complexes (80 μg/400 μg/4 mg). The tumour volumes were measured every 2 days. Once the tumour volume reached 2500 mm^3^, the euthanasia procedures of mice were performed under the standard protocol. The tumours and organs of the mice were excised, fixed with paraformaldehyde, and examined by microscopic haematoxylin and eosin (H&E) staining.

### H&E staining

The tumours and organs of the mice were embedded in paraffin wax after dehydration. Then, the tissue sections (4 μm/section) from paraffin-embedded blocks were collected on clean glass slides and dehydrated for 30 min at 60 °C. The tissue slides were further deparaffinized, rehydrated, and stained with Mayer’s haematoxylin and eosin Y solution for 3 min. Finally, the tissue slides were mounted with mounting media and photographed by microscopy.

## Statistical analysis

The data represent the mean ± standard deviation (SD) of two or three independent experiments. Statistical analysis was performed by Student’s *t*-test or analysis of variance (ANOVA) with Bonferroni or Games-Howell test and were calculated with IBM SPSS software (IBM Corp., Armonk, NY, USA). The significant differences are labelled with *for P < 0.05.

## Supplementary information

**Additional file 1: Figure S1.** In vitro transfection ability of the LPPC/DNA/RBDV complexes. Different concentrations of RBDV were encapsulated by LPPC/DNA, which was transfected into B16-F10 cells, and the cells were analysed for (A) the transfection efficiency and (B) the mean fluorescence intensity by flow cytometry. LPPC, which encapsulated with RBDV, were all complexed by PEG. The data represent the mean ± SD (n = 2).** Figure S2.** In vivo the effects on tumour growth inhibition of RBDV or IgG1 Fc. Female C57BL/6 mice (6-8 weeks of age) were subcutaneously inoculated with 1 × 10^6^ cells in 100 mL of PBS. When the average tumour volume reached 30 mm^3^, the mice were intravenously (i.v.) injected with LPPC/RBDV-IgG1 Fc plasmid /RBDV-IgG1 Fc protein or other groups. ▼ Means the day of complex injection. (n = 3). LPPC, which encapsulated with RBDV or IgG1 Fc, were all complexed by PEG.

## Data Availability

The datasets used and/or analysed during the current study are available from the corresponding author upon reasonable request.
