# Supplementary material for: Lipoplex-based targeted gene therapy for the suppression of tumours with VEGFR expression by producing anti-angiogenic molecules
Source: J Nanobiotechnology. 2020 Apr 9;18:58. doi: 10.1186/s12951-020-00610-9 (PMC7144055; doi:10.1186/s12951-020-00610-9)
Supplement: Supplementary file 1 — Additional file 1: Figure S1. In vitro transfection ability of the LPPC/DNA/RBDV complexes. Different concentrations of RBDV were encapsulated by LPPC/DNA, which was transfected into B16-F10 cells, and the cells were analysed for (A) the transfection efficiency and (B) the mean fluorescence intensity by flow cytometry. LPPC, which encapsulated with RBDV, were all complexed by PEG. The data represent the mean ± SD (n = 2). Figure S2. In vivo the effects on tumour growth inhibition of RBDV or IgG1 Fc. Female C57BL/6 mice (6-8 weeks of age) were subcutaneously inoculated with 1 × 10^6^ cells in 100 mL of PBS. When the average tumour volume reached 30 mm3, the mice were intravenously (i.v.) injected with LPPC/RBDV-IgG1 Fc plasmid /RBDV-IgG1 Fc protein or other groups. ▼ Means the day of complex injection. (n = 3). LPPC, which encapsulated with RBDV or IgG1 Fc, were all complexed by PEG. [file 12951_2020_610_MOESM1_ESM.docx]

Additional file 1

Figure S1

**
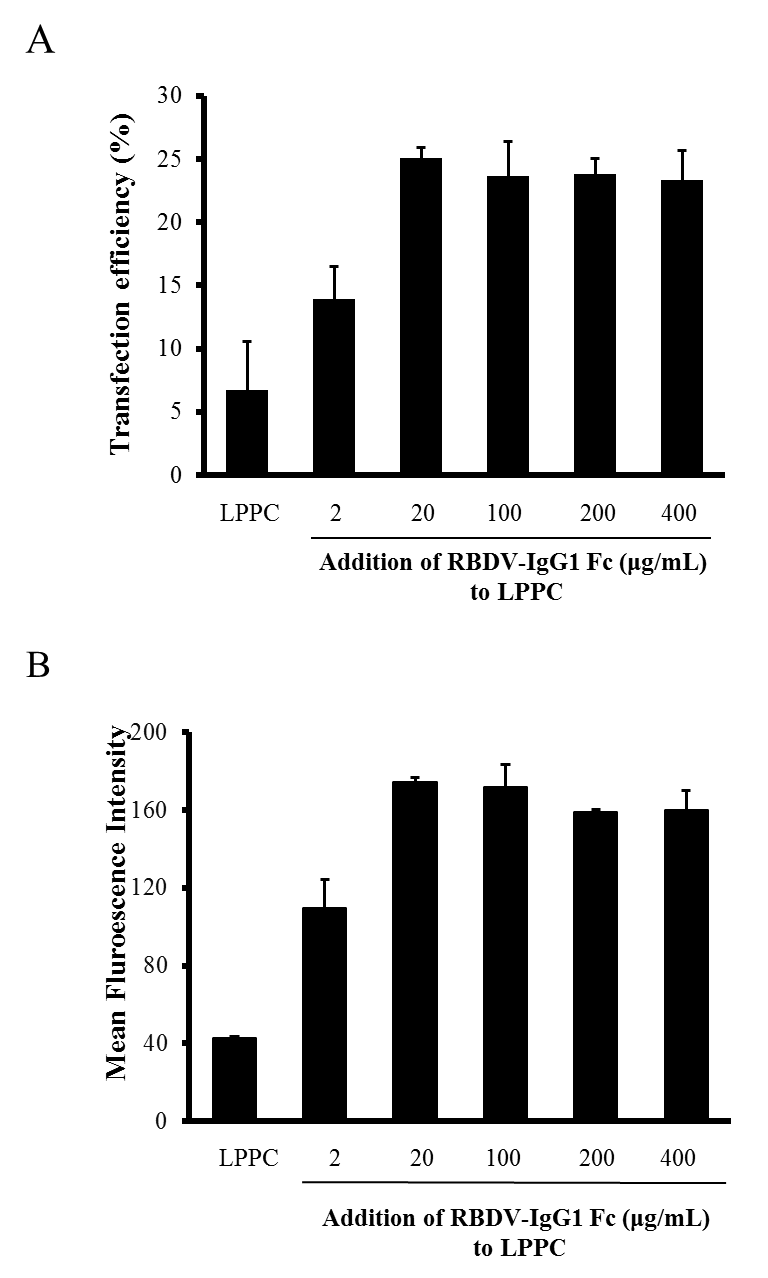
**

**Figure S1. *In vitro* transfection ability of the LPPC/DNA/RBDV complexes.**

Different concentrations of RBDV were encapsulated by LPPC/DNA, which was transfected into B16-F10 cells, and the cells were analysed for (A) the transfection efficiency and (B) the mean fluorescence intensity by flow cytometry. LPPC, which encapsulated with RBDV, were all complexed by PEG. The data represent the mean ± SD (n=2).

Figure S2

**
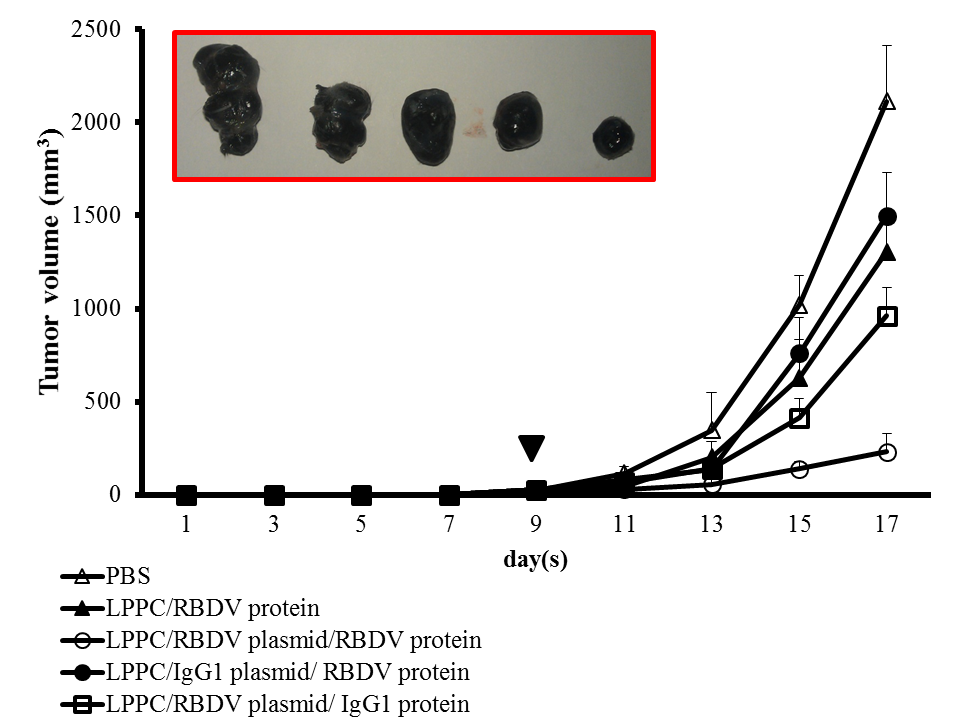
**

**Figure S2. *In vivo* the effects on tumour growth inhibition of RBDV or IgG1 Fc.**

Female C57BL/6 mice (6-8 weeks of age) were subcutaneously inoculated with 1 × 10^6^ cells in 100 mL of PBS. When the average tumour volume reached 30 mm^3^, the mice were intravenously (i.v.) injected with LPPC/RBDV-IgG1 Fc plasmid /RBDV-IgG1 Fc protein or other groups. ▼ Means the day of complex injection. (n= 3). LPPC, which encapsulated with RBDV or IgG1 Fc, were all complexed by PEG.
